# The Origins of Ploem's Filter Cube: A Pandora's Box

**DOI:** 10.1111/jmi.70137

**Published:** 2026-06-24

**Authors:** Jack Gordon, Kaleah Balcomb, Vinod Maseedupally, Maitreyee Roy

**Affiliations:** ^1^ School of Optometry and Vision Science University of New South Wales Sydney Australia; ^2^ Department of Neuroscience Faculty of Medicine and Health School of Medical Sciences University of Sydney Camperdown Australia; ^3^ Brain and Mind Centre University of Sydney Camperdown Australia

## Abstract

Ploem's filter cube, central to modern epifluorescence microscopy, integrates three matched optical components: an excitation filter, an emission filter, and a dichroic mirror. Together, these elements enable the separation of excitation and emission light paths, producing high‐contrast fluorescence images. Despite their importance, the origins of these optical filters and the concept of a matched filter set remain unattributed. This review traces the development of these principles to discoveries made half a century before the fluorescence microscope. Through a narrative historical analysis, we identify George Stokes, Wilhelm Haidinger, and David Brewster as key contributors whose studies of light, colour, and polarisation established the foundations of fluorescence filtering. We also show that the matched filter concept can be dated to 173 years ago and that early dichroic films predated Ploem's cube by more than a century. By uncovering these origins, this work highlights how early optical discoveries shaped the design of modern fluorescence microscopy.

## LAY DESCRIPTION

Fluorescence microscopy utilises three main optical filters: the excitation filter, the emission filter, and the dichroic filter. In the literature, there is no current attribution as to who invented these filters. They also operate together using a core concept (a matched filter set) which occasionally has not been understood by many scientists seeking to construct their own fluorescence microscopes. To provide practical instruction to those seeking to understand how fluorescence microscopes work, we have undertaken a narrative review tracing the invention of a matched filter set to a date of discovery 173 years ago, 50 years before the invention of the fluorescence microscope. We have also detailed how these scientists produced the earliest examples of thin dichroic films more than a century before these designs of excitation filters, emission filters and dichroic filters were all combined to form the workhorse of present‐day fluorescence microscopy, the epifluorescence filter cube.

## 1 INTRODUCTION

### 1.1 Epifluorescence filter cubes

Fluorescence microscopy is primarily applied in histopathology, immunology and drug target discovery, but is also used in sister fields, like neurosurgery, dentistry, ophthalmology and even forensic work. These fields all benefit from the optical filters developed in fluorescence microscopy. The workhorse of fluorescence microscopy is the epifluorescence widefield microscope and epifluorescence filter cube, first introduced by Evgenii Mikhailovich Brumberg (1907–1986) and later developed by Johan Sebastiaan Ploem (born 1927).^1^, ^2^, ^3^, ^4^, ^5^ This filter cube integrates three main components: the excitation filter, emission filter and dichroic mirror, otherwise known as a beamsplitter (Figure 1).

**FIGURE 1:**
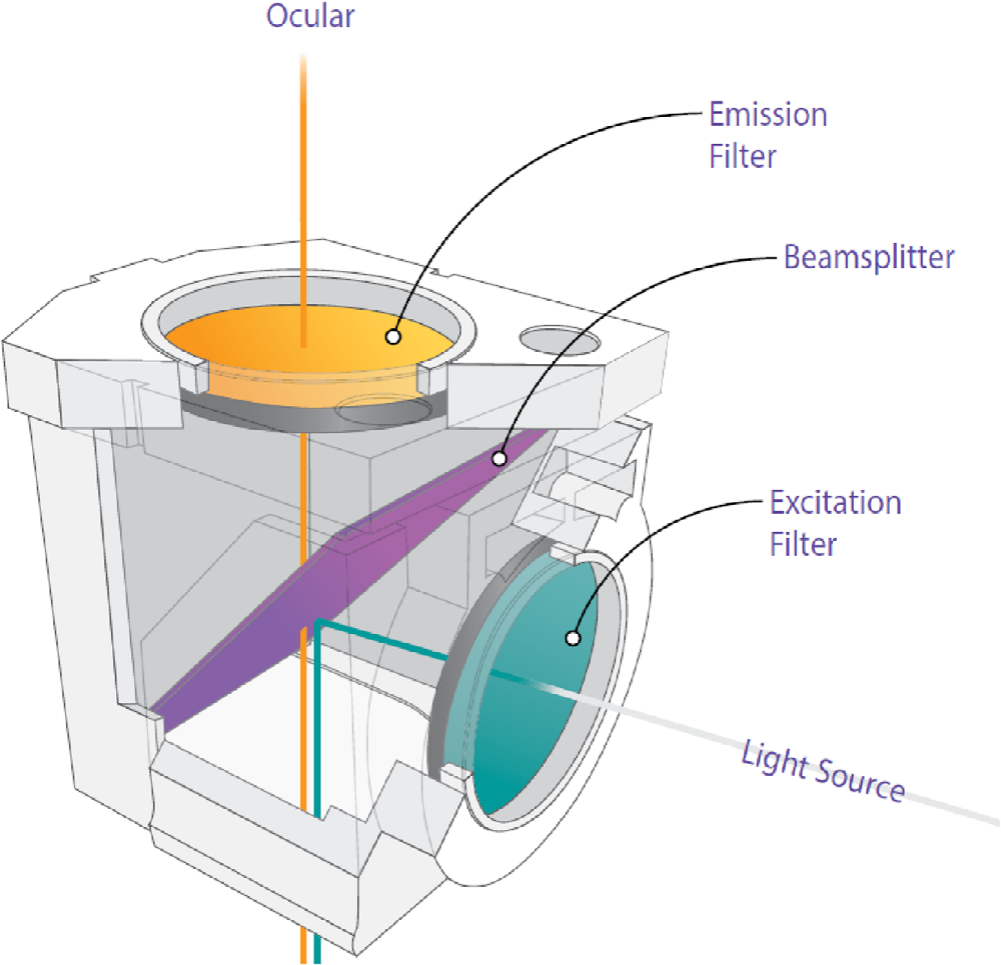
Typical placement of the excitation, emission and dichroic filter or Beamsplitter within the filter cube. Reproduced with permission courtesy of Chroma Technology.^6^

The excitation and emission filters of this epifluorescence filter cube have been adapted to aid dental examination, to fit contact lenses, to highlight clinical details in the anterior and posterior segments of the eye, and even to aid fluorescence‐guided neurosurgery.^7^, ^8^, ^9^, ^10^

In these adapted contexts, the excitation filter selectively transmits wavelengths from the light source capable of exciting fluorescence within the specimen. The excitation light is then directed by the dichroic mirror through the microscope objective onto the fluorescent specimen, after which the emitted fluorescent light is collected by the same objective lens. The dichroic mirror, otherwise known as a beamsplitter, reflects the shorter wavelength excitation light toward the specimen while permitting the longer wavelength fluorescent emission to pass toward the eyepiece or detector. The emission filter further blocks residual excitation and scattered light, ensuring that primarily fluorescent emission contributes to the final image. Together within the filter cube (Figure 1), the excitation, emission and dichroic filters form a matched filter set capable of producing high‐contrast fluorescence images through efficient separation of excitation and fluorescent light.

Additionally, dichroic mirrors facilitate episcopic illumination, a technique in which the fluorescent specimen is illuminated and observed through the same objective lens.^6^ Using episcopic illumination, a dichroic filter alone can reduce the amount of excitation light relative to observed fluorescence by as much as 10^−5^ or 10^−6^, which is similar to the attenuation achievable by some emission filters.^6^ Yet when excitation, emission and dichroic filters are used in conjunction within a filter cube (Figure 1) their synergistic effect can reach attenuations as low as 10^−11^.^6^

The origins of these components have yet to be formally attributed in the literature to date, despite modified versions of the excitation and emission filter featuring in modern health sciences.^7^, ^9^, ^11^, ^12^, ^13^, ^14^ The implementation of matched filters within these instruments historically has been poorly understood^9^ having been used either empirically,^15^ by chance^8^ or not at all.^9^, ^16^, ^17^ The introduction of matched filters to an imaging setup yields vast improvements in image quality, which is often mistakenly credited as a moment of rediscovery.^12^, ^18^, ^19^, ^20^, ^21^, ^22^, ^23^ Ambiguity also exists regarding the origins of the dichroic mirror, sometimes attributed to Brumberg or Ploem.^6^, ^24^, ^25^ This review aimed to identify the scientific minds who contributed to each of these elements, and credit those who have previously gone unrecognised.

### 1.2 Origins of the emission filter

#### 1.2.1 Early fluorescence microscopy

In fluorescence microscopy, the emission filter serves to transmit only desirable wavelengths of fluorescent light from the specimens to the eyepiece or detector, while blocking unwanted excitation light ‘noise’. This generally improves contrast of the fluorescent specimen and allows multiplexing of multiple fluorophores within a single specimen; a technique frequently applied in biology and neuroscience to assess the organisation and colocalisation of cellular components. Yet at the beginning of the 20th century, early fluorescence microscopes were designed entirely without emission filters,^1^, ^26^ possibly due to configurations in which they were simply not necessary. Wilhelm Röttgen (1845–1923) demonstrated that invisible X‐rays could elicit fluorescence in a sensitive platinum cyanide plate without the use of an emission filter.^27^ Around the same time, Robert Wood (1868–1955) implemented a novel ultraviolet light excitation filter utilising nitroso‐dimethylaniline dye that was ‘…wholly opaque to visible light’,^28^, ^29^ which was later implemented in early fluorescence microscopes to examine fluorescent substances without the need for emission filters.^1^, ^26^ Philipp Ellinger (1887–1952) reflected on this use of invisible ultraviolet excitation; ‘…the contrast between the light fluorescent patches and the dark non fluorescent patches became more marked with this method than with the usual microscopy in transmitted white light’.^30^

#### 1.2.2 Ultraviolet light microscopy

The contrast produced by invisible ultraviolet light illumination offered the chance for a serendipitous discovery by August Köhler (1866–1948) in 1904.^31^ Inspired by earlier work relating increased microscopic resolution to shorter wavelengths of light,^32^ Köhler constructed the first ultraviolet light microscope using a 275 nm cadmium spark light source, which produced images using a photographic plate sensitive to invisible ultraviolet light.^33^, ^34^ Prior macroscopic experiments had demonstrated the visualisation of unseen ultraviolet light by a sensitive fluorescent substance.^26^, ^35^ Köhler applied this concept to create his ‘fluorescent seeker system’ (Figure 2), which enabled focussing of otherwise imperceptible light through the microscope objective.^33^, ^34^, ^36^ This arrangement unexpectedly enabled the visualisation of a fluorescent substances against a black background, leading to Köhler's serendipitous observation of microscopic autofluorescence in biological tissue.^1^

**FIGURE 2:**
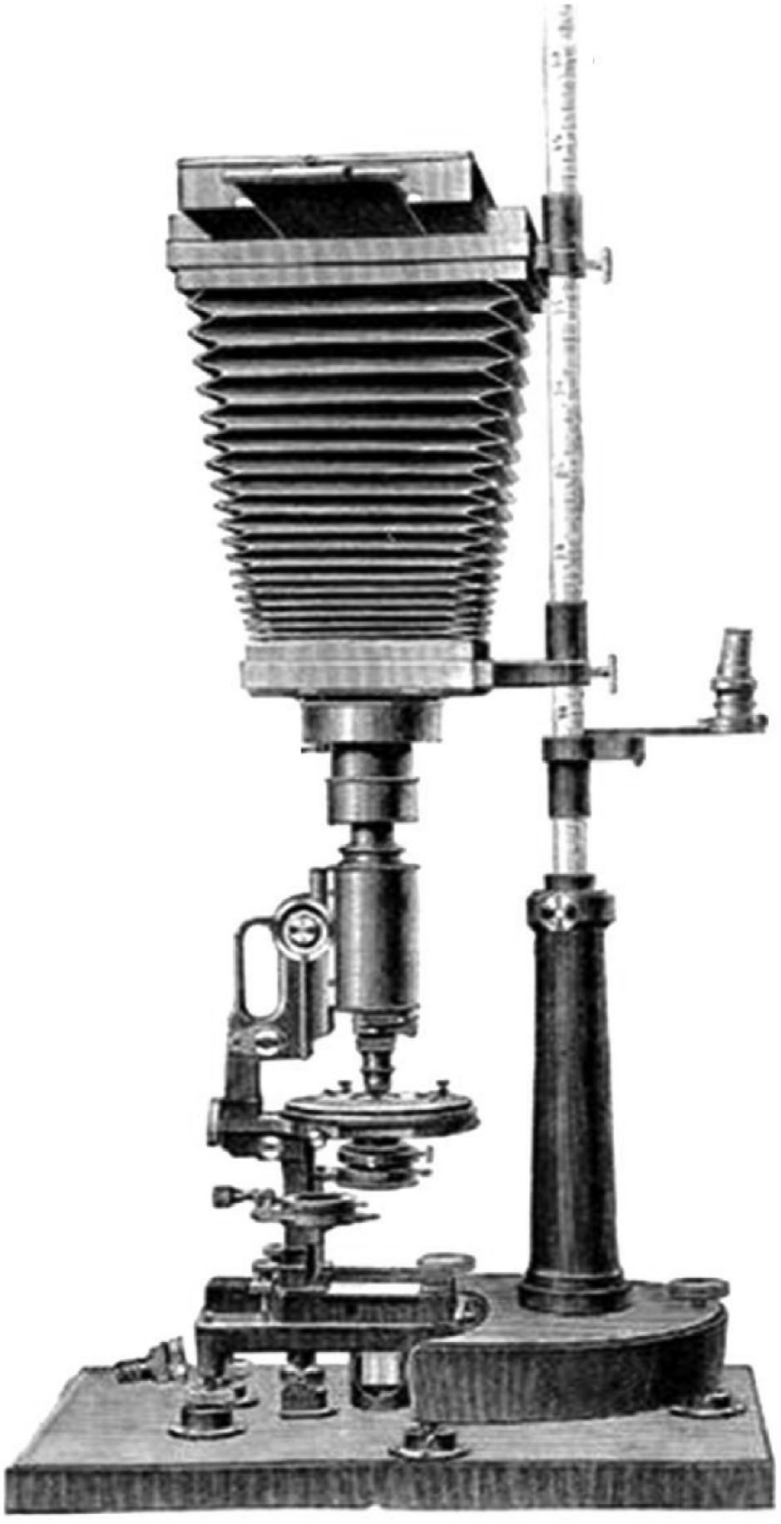
Köhler's ultraviolet microscope included a ‘fluorescent seeker system’ whereby a fluorescent focusing plate could be replaced by a photographic plate for image capture.^31^

Köhler's 275 nm cadmium spark could only dimly excite fluorescence^30^ and so was replaced by an ‘iron carbon’ arc light introduced by Heinrich Lehmann.^1^, ^29^, ^37^ The addition of visible light, while greatly improving fluorescent signal, came at the cost of high visibility of background light to the microscopist. Lehmann described this as ‘glare’^38^ because it reduced the contrast of a given fluorescent specimen^30^ (Figure 3).

**FIGURE 3:**
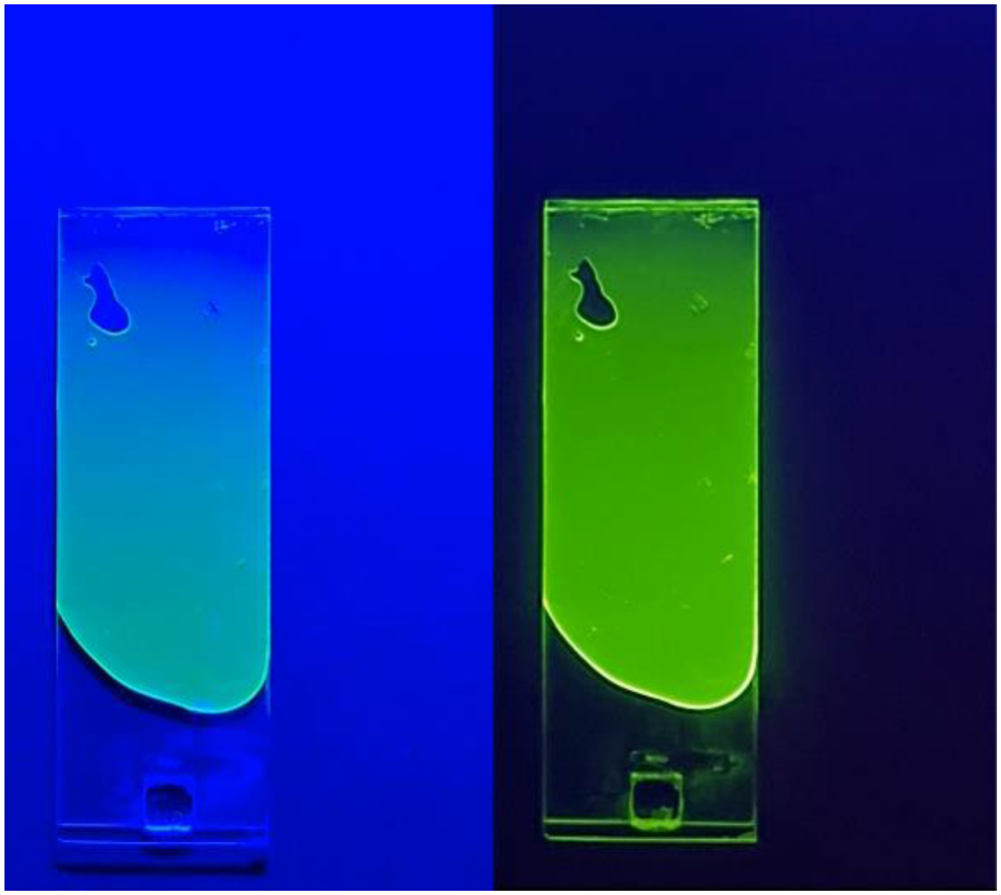
Fluorescein under visible light filtered by cobalt blue glass, with no emission filter (left), or with a Wratten No. 12 emission filter (right), which significantly reduces blue background noise.

#### 1.2.3 Darkfield fluorescence microscopy

The glare observed by Lehmann when using visible light would have been an appropriate context to introduce the emission filter. Instead, an entirely different solution was presented by Köhler and Henry Siedentopf (1872–1940), who ingeniously incorporated a darkfield condenser lens into their cadmium spark ultraviolet microscope in 1908 during a vacation course on scientific microscopy.^30^, ^37^, ^39^ Recognising the ability of this design to reduce the visible light glare with Lehmann's new light source, Oskar Haimstadt (1878–1948) used both a darkfield condenser and Lehmann's light source in the Reichart company's fluorescence microscope to further reduce visible light glare.^1^, ^24^, ^39^, ^40^, ^41^ This timely innovation leveraged the newly recognised Lambertian properties of fluorescent emissions;^38^, ^42^ unlike the directional excitation light employed by the darkfield condenser lens, the fluorescent emission ‘would expand out in all directions’ and thus be collected by the objective lens while the ultraviolet excitation light was simultaneously directed away from the objective (Figure 4). However, the darkfield condenser failed to the remove scattered excitation light from the specimen,^24^ and limited the amount of possible excitation light, resulting in a fluorescent image which still appeared dim to the observer.^38^

**FIGURE 4:**
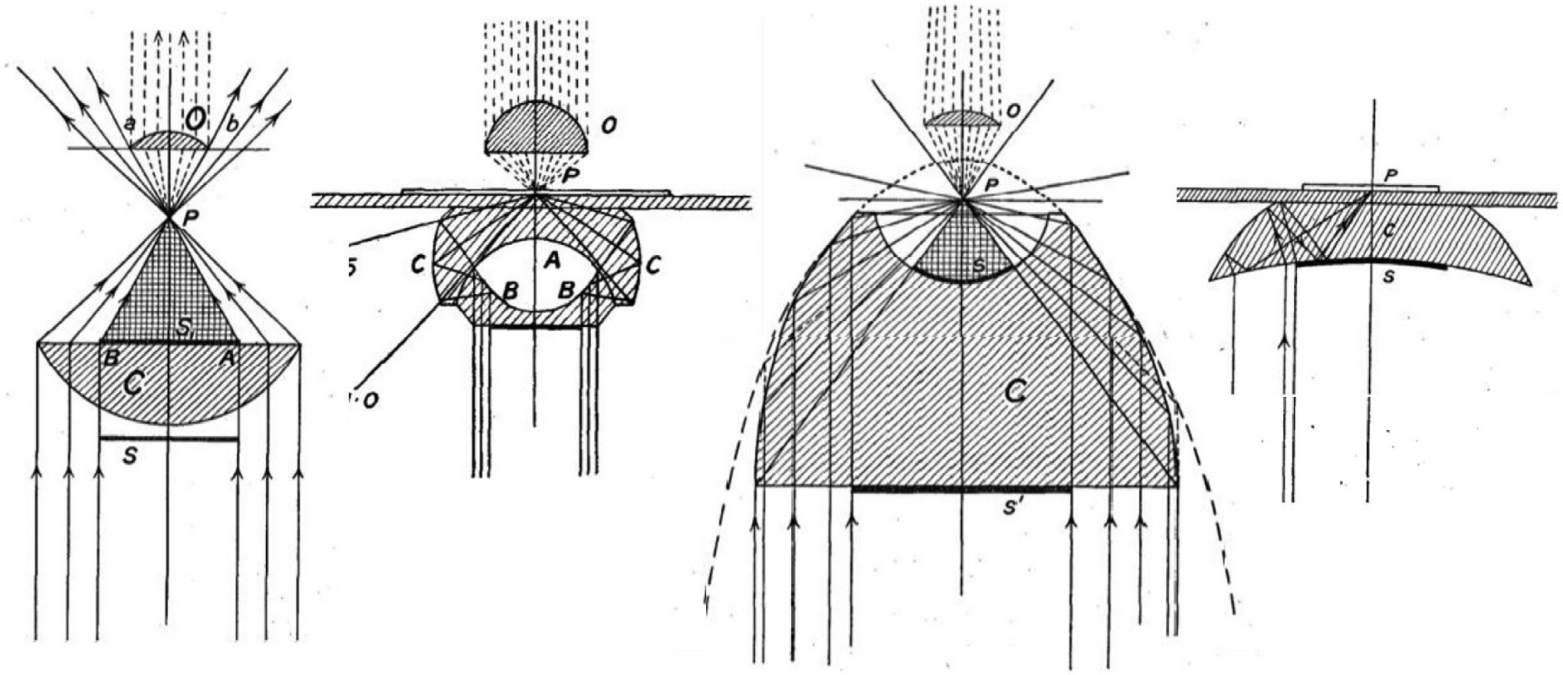
Schematic diagrams of dark field microscopy setups drawn by Aisley. Reproduced with permission.^43^

#### 1.2.4 The Euphos coverslip

Lehmann had come up with an alternative solution for the visible light glare, an emission filter, in the form of a coverslip impermeable to short wavelength light.^1^, ^30^, ^38^ He specified that this emission filter or ‘trap filter’ should be of sufficient thickness to filter out the ‘primary radiation’ (excitation light),^30^ and that it should be ‘located in a sliding plate between the objective lens and the microscope’.^30^ He also stated that this filter ‘passes visible light without altering colours’ and ‘does not itself fluoresce’^38^ (Figure 5).

**FIGURE 5:**
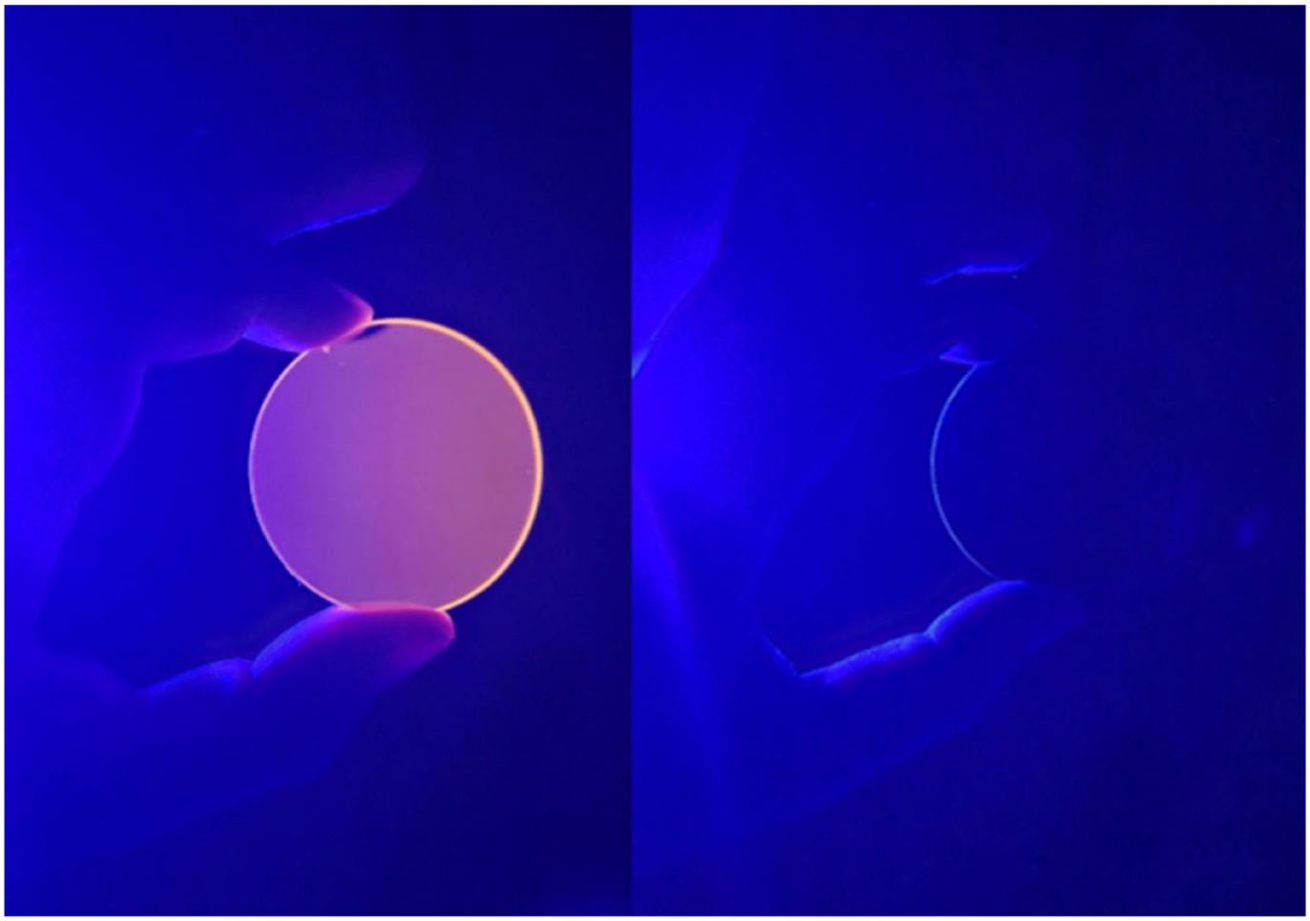
A 545 Schott emission filter exhibits its own fluorescence (left), while the Wratten No. 12 filter shows an absence of its own fluorescence (right) under blue LED light.

**FIGURE 6:**
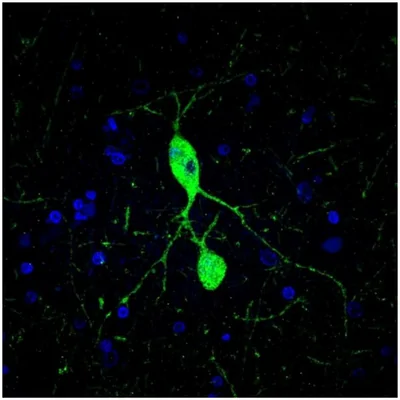
A parvalbumin‐positive neuron illustrates the concept of a fluorescent substance being ‘luminous in a dark field’, provided by Kaleah Balcomb.

Having initially used heavy flint glass,^37^ Lehmann switched to the thinner ‘Euphos’ glass coverslip due to its lack of fluorescence.^38^ This Euphos filter was a commercially available filter capable of blocking ultraviolet light.^44^ Many trap filters became available following Lehmann's work,^30^ and his colleague Paul Metzner subsequently placed this barrier filter at the ocular of later versions of fluorescence microscopes (See Figure 6).^1^

#### 1.2.5 George Stokes: The true pioneer of emission filters

It might be intuitive to attribute the invention of the emission filter to Lehmann,^30^ given the level of detail in his specification of filter requirements. Yet the first use of an emission filter was not the Euphos coverslip, but rather a glass of white wine,^45^, ^46^ a mirror of burnt silver,^47^ or a solution of potassium dichromate^1^ implemented by George Stokes (1819–1903) some 60 years prior. Stokes had also informally used cobalt blue glass as an emission filter when, holding it to his eye after a period of dark adaptation, he was able to view a distinct red band emanating from fluorescent quinine.^35^ Stokes’ work may have also formed the basis for the excitation filters used by Wood and Lehmann: Stokes had already made use of flint glass as an emission filter,^48^ and both Lehmann and Wood expressed a similar preference for darker cobalt blue glass filters,^8^, ^26^ and were aware that cobalt glass transmits a red spectrum that is absent in a filter composed of copper sulphate solution. It is likely because of this complementary property that copper and cobalt formed the basis for Wood and Lehmann's ultraviolet light filter combinations.^28^, ^29^, ^49^

#### 1.2.6 Matched filters

In addition to his pioneering use of emission filters, Stokes additionally outlined the conceptual basis of matched excitation and emission filters in 1852,^50^ which continues to form the basis of modern epifluorescence microscopes.^47^ He specified that the light transmitted by an excitation and emission filter should be mutually exclusive, or ‘strictly complementary’, detailing how these filters should work together:

Imagine two such media prepared, of which the second was opake with regard to those rays of the visible spectrum with regard to which the first was transparent, and vice versd. … if both media were held in front of the eye no light would be perceived. … But if any of the objects [placed between the media] had the property of emitting rays of one refrangibility under the influence of rays of another [were fluorescent], it might happen that some of the rays so emitted were capable of passing through the second medium, in which case the object would appear luminous in a dark field.^47^ (Figure 6).

Unfortunately, Stokes’ work appears to have been overlooked^30^, ^38^ as it was fifty years before Köhler's work on fluorescence microscopy, and more than a century before matched filters were commercialised in 1975.^18^

The pairing of copper sulphate and a yellow glass coloured by silver came closest to Stokes’ ideal filter set, and he reported striking results when a piece of canary glass (uranium) was used between these two media.^47^ Stokes remarked that even faint fluorescence of thin films became visible after implementing these matched filters, reiterated 60 years later by Lehmann when he discovered the same effect.^38^, ^47^ Stokes also proposed that these matched filters could serve as a test for weak fluorescence. To illustrate a negative result, Stokes invited his readers to hold the paired excitation and emission filters before their eyes to ensure they saw darkness, since no light other than fluorescent light could pass through Stokes’ arrangement^47^ (Figure 7).

**FIGURE 7:**
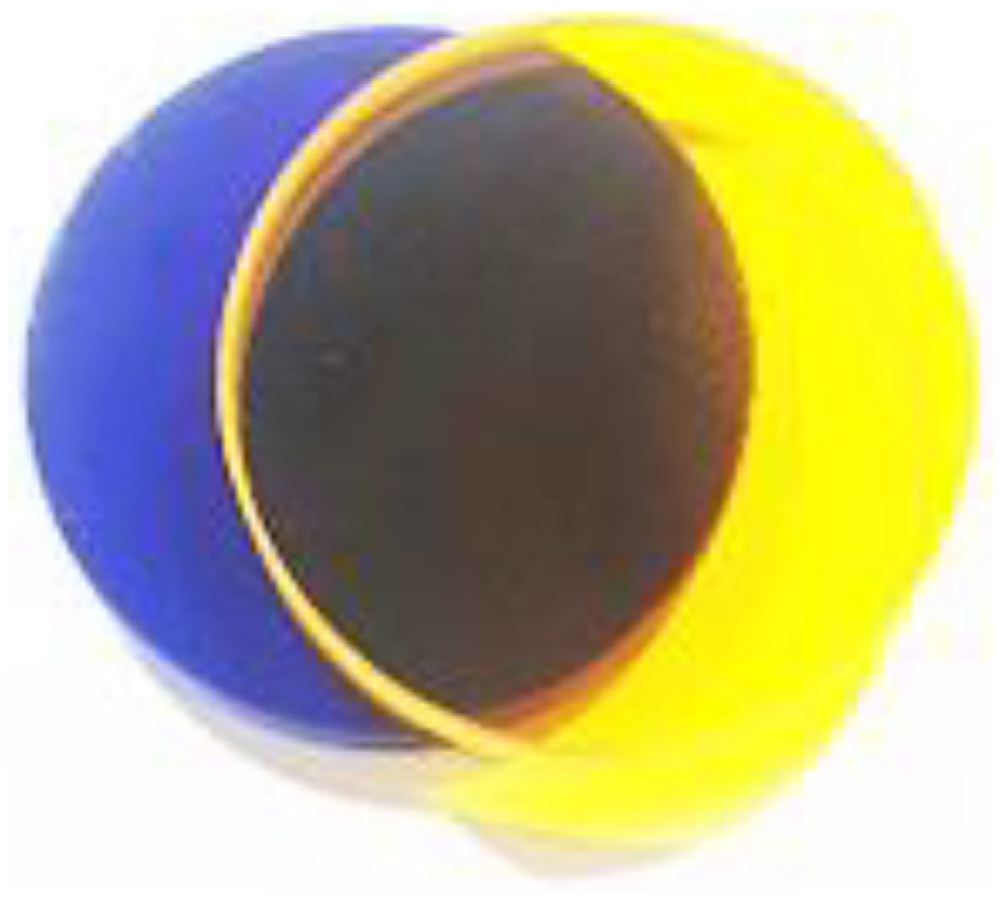
A set of matched filters exhibits mutually exclusive transmission.

Stokes used this test for fluorescence to determine whether quinine medication was genuine,^51^ as falsified preparations of the malaria treatment lacked fluorescent properties.^52^ Unfortunately the test was difficult to perform, and Stokes acknowledged the difficulty of finding appropriate media to use as matched filters, pointing out ‘… all we can do is to select from among those which occur in nature … [because] it is not possible to obtain media [optical filters] which are strictly complementary’.^47^, ^51^ Stokes’ matched filters consequently fell from public knowledge, albeit with one silver lining; it was the study of these microscopic crystals of quinine that led Stokes to elucidate the behaviour of the first dichroic mirror.^51^, ^53^, ^54^, ^55^

### 1.3 Synthesis of the dichroic mirror

#### 1.3.1 Quinine

It was around this time that a series of experiments upon quinine provided confusing and contradictory results. Quinine is a complex molecule possessing fluorescent, chiral and dichroic properties,^51^, ^53^ all of which affected its interaction with polarised light in solution.

These optical properties initially posed a quandary for scientists such as Sir John Herschel (1792–1891), who supposed that quinine's blue fluorescence might be the result of reflection from suspended particles within a solution.^56^ Herschel therefore expected to observe the well‐known Tyndall phenomenon, whereby light reflected by suspended particles was observed to be linearly polarised. Similar effects had been documented in reflected firelight,^57^ in sunlight reflected from water droplets in clouds,^58^ or particles in comet tails.^59^ Stokes noticed Herschel's hesitancy in reaching this conclusion, and drew attention to Herschel's observations that fluorescent quinine lacked the expected polarisation.^35^, ^56^

He conceived it to be due to coloured particles held in suspension. But … a whole or a great part of the dispersed beam was unpolarised, where as a beam from suspended particles might be expected to be polarized by reflexion.^35^

Although Stokes later hinted that experimentation with platinum cyanide had revealed ‘… a new and remarkable feature, which consists in the polarisation of the fluorescent light’,^47^ he provided no further details about this experimentation, and it would be another 50 years before Robert Wood suggested that fluorescent light emission could somehow retain or increase the polarisation of it's exciting light.^60^ Yet this mystery expanded further when David Brewster introduced quinine to linearly polarised excitation light, and observed that the resulting fluorescence was polarised in all directions.^61^ Perplexed by this result, Brewster described a theory of ‘an infinite number of doubly refracting crystals having their axis of refraction lying in every possible direction’.^61^

Fortunately, an answer to this quandary was forthcoming, as Stokes had shown quinine's fluorescence was dependent upon pH^47^, ^48^: acidic solutions made the fluorescence of quinine vanish.^62^ Apollinaire Bouchardat (1809–1886) and Louis Pasteur (1822–1895) used this non‐fluorescent acidic solution of quinine to demonstrate quinines’ chirality by rotating the polarisation of light, putting forward this property of the optically active quinine as an additional test of malaria medication legitimacy.^51^ The test could also distinguish quinine's isomer, quinidine, which produced a polarity deviation to the right, while quinine rotated polarisation to the left.^63^, ^64^

While questions remained on how best to separate the effects of fluorescence, chirality and the Tyndall effect when observing quinine, a fourth phenomenon appeared: the dichroism exhibited by anisotropic crystals, closely associated with the polarisation of light.^54^

#### 1.3.2 Dichroism of thin anisotropic crystal plates

Quinine's polarising nature was later studied by William Herapath (1820–1868), who noted that after a dog had been fed quinine, it's urine produced crystals that polarised light when mixed with iodine.^65^ Herapath's work involved distinguishing a green dichroic reflection from the blue fluorescence of quinine solution, demonstrating a phenomenon whereby certain wavelengths of the incident light were reflected while other wavelengths were transmitted. Herapath additionally discovered that when crystals were precipitated out of solution, they transmitted a deep garnet red light, but when they were dissolved in chloroform and mounted in Canada Balsam they maintained their fluorescent appearance.^53^ Herapath further stated that under certain conditions a solution could exhibit both dichroic and fluorescent properties simultaneously.^53^ Wilhelm Haidinger (1795–1871) also became interested in Herapath's precipitated quinine crystals, recognising that they displayed dichroic properties, and termed the iodoquinine sulphate ‘Herapathite’ in acknowledgement.^53^, ^55^ After formation of crystal plates 1/300th of an inch in thickness, the plates transmitted light of one polarisation but were ‘opaque and black’ to perpendicularly oriented polarisation, ‘however thin the plates may be’.^55^ The crystal transmitted light of a deep blood red colour, while simultaneously reflecting green light of opposite polarisation. Haidinger also observed that in slightly thicker sections of the plate, crystals with overlapping crossed polarisations would cause no light to be transmitted.^55^ This work later prompted Edwin Land (1909–1991) to develop a polarising film from the quinine salt in the 20th century, resulting in the world's first synthetic polariser and polaroid camera^65^ Land later reflected upon this discovery:

Then came the most exciting single event in my life. The suspension of herapathite crystals was placed … (aside a) … magnet which could produce about 10,000 gauss. Before the magnet was turned on, the Brownian motion caused the particles to be oriented randomly so that the liquid was opaque and reddish black in color. When the field was turned on‐and this was the big moment‐slowly and somewhat sluggishly the cell became lighter and quite transparent; when we examined the transmitted light with a Nicol prism, it went from white to black as the prism was turned.^65^

#### 1.3.3 Dichroism in fluorescent microscopy

Although macroscopic tourmaline crystals had been known to exhibit dichroism for some time, the discovery of dichromatism in thin films of precipitated crystals is attributed to Brewster and Haidinger.^54^, ^66^, ^67^ To confirm their work, Stokes viewed reflected and transmitted light from dichroic films formed on plates of glass via evaporation through a prism to determine that these lights were complementary in colour and wavelength.^54^ For a crystallised film of permanganate of potash, Stokes observed 5 distinct bands that were not transmitted, matching their wavelengths with four distinct reflected bands, with the remaining fifth band being faint and indistinct, confirming Haidinger's assertion of their dichroic behaviour.^54^ Lord Rayleigh (1842–1919) remarked upon the sharpness of termination of the bands of reflected light from dichroic substances.^68^ The practice of decomposing thin films of dichroic salt continued^69^, ^70^ and Haidinger made similar observations on Mausite^71^ and others later on the picrate of Manganese.^66^ Dichroism resulting from thin films of metal oxides deposited by electrolysis were studied in 1891,^72^ observations of dichroism in thin sheets of gold were made in 1894,^73^ and the dichroic behaviour of thin films of metal caught the attention of many researchers at the turn of the 20th century.^74^ Oxides with dichroic properties were deposited onto glass using sublimation, followed by evaporation, also known as vacuum deposition.^75^, ^76^, ^77^

#### 1.3.4 Synthetic dichroic mirror

The development of the synthetic dichroic mirror emerged through the study of natural optical phenomena, particularly optical anisotropy, thin‐film interference, and multilayer reflective structures. Aligned, repeating arrangements of anisotropic molecules within crystal lattices, and stretched synthetic silk,^78^ as well as liquid suspensions of molecules oriented via laminar flow^79^ or magnetism^80^, ^81^ produced dichroic reflections. Earlier attempts to produce synthetic dichroic filters attempted to orient molecules using transparent media^82^ either by colouring crystal lattices or stretched cellulose with dichroic dyes.^83^, ^84^

However, Stokes had recorded observations of ‘twinning’ within crystal of chlorate of potash,^85^ discovering an additional mechanism underpinning natural and synthetic dichroic mirrors today.^86^, ^87^ Stokes discovered that several planes of dichroic crystals were able to combine their reflective properties to produce more vivid dichroic reflections. Lord Rayleigh later revisited Stoke's observations, calculating that a small 0.14 mm crystal plate held about 700 parallel reflecting planes which together were responsible for a vivid purple colour.^85^

It was later recognised that this multilayer arrangement could make use of thin film interference: ‘By evaporating alternate layers of high and low‐index insulators on glass, it is possible to produce a surface having transmission and reflection characteristics and no appreciable absorption’.^85^ The first use of a multilayer film is attributed to Cartright, who was aware of Stoke's observations,^88^ although primary sources seem no longer available.^89^, ^90^, ^91^ Widdop reports reflecting over 90% of a specified wavelength using this method,^92^ a concept which Stokes himself imagined would be exceptionally useful: ‘Conceive that we had the power of producing at will media which should be perfectly opaque with regard to rays belonging to any desired regions of the spectrum…’.^47^

Despite these technological advances, dichroic filters were first introduced in microscopy via thin films of stretched cellulose plastic imbued with dichroic dyes in phase contrast microscopy,^93^ followed by Brumberg's pioneering combination of use of episcopic and darkfield illumination, introducing the dichroic filter to fluorescence microscopy.^6^ In 1967 Ploem would publish work combining this approach with Stoke's matched filters to create the epifluorescence filter cube, albeit synthesised with thin film interference filters rather than anisotropic materials or absorption filters. The process of vacuum deposition of dielectric materials aided Brumberg and Ploem almost a century after Stokes’ work^94^, ^95^ when these authors integrated dichroism into epifluorescence filter cube we know today.^5^, ^24^

### 1.4 Super resolution fluorescence microscopy

There is a certain irony when considering Köhler's original intention, to improve upon the diffraction limited resolution of the microscope, as his work would lead to fluorescence microscopy, and later facilitate super resolution fluorescence microscopy.^31^ In 1872, Helmholtz had postured that microscopic objects ‘would be more easily discernible if the microscopic objects themselves were to emit light’,^30^ foreshadowing an accidental discovery by Bailey et al.^96^ This group had been attempting to use a technique known as total internal reflection fluorescence (TIRF)^97^ to selectively excite fluorescent molecules at the bottom of a fluorescent labelled cell. In doing so, a simple error had been made by mismatching the refractive index of the mounting medium with that of coverslip. It was later understood that this mismatch in refractive index allowed reflection to occur at the upper surface of the coverslip, producing constructive and destructive interference within the excitation light passing through the cell. Unbeknownst to the researchers, a standing wave formed from the excitation light extended along the length of the cell, producing periodic sinusoidal bands of illumination.

…and we saw when we pressed on the stage, that there were zones of fluorescence that moved up and down through the cell.^98^

This observation contributed to the development of a microscope with axial resolution exceeding the diffraction limit established by Abbe,^32^, ^96^ posthumously validating Helmholtz’ speculation. As one of the first examples of super resolution fluorescence microscopy in the 1990s, this and other discoveries led to an explosion of super resolution fluorescence microscopy techniques, including stimulated emission depleted (STED) microscopy and single‐molecule localisation methods such as photoactivated localisation microscopy (PALM) and stochastic optical reconstruction microscopy (STORM), for which Eric Betzig, Stefan Hell, and William Moerner were awarded the Nobel Prize.^99^

## 2 CONCLUSION

The scientific developments underlying the Ploem's filter cube span many decades and reflect the contributions of numerous researchers. Although the implementation of an emission filter is credited in the literature to Lehmann and Metzner,^30^ it was Stokes who first employed an emission filter to attain a black background in which a fluorescent substance appeared ‘*luminous in a dark field’*. Indeed, had Stokes’ work on matched filters been more widely recognised, the field of fluorescence microscopy might have advanced at a greater pace. Alongside Herapath, Haidinger and Brewster, Stokes also conducted important research demonstrating the ability to create thin dichroic films, a concept which has since progressed as our understanding of methodology advanced. The advent of thin film interference filters has allowed the realisation of Stokes’ concept of matched excitation and emission filter sets, a principle that remains central to the optical design of contemporary fluorescence microscopes.
